# Sphingolipids in Obesity and Correlated Co-Morbidities: The Contribution of Gender, Age and Environment

**DOI:** 10.3390/ijms20235901

**Published:** 2019-11-24

**Authors:** Enrica Torretta, Pietro Barbacini, Nasser M. Al-Daghri, Cecilia Gelfi

**Affiliations:** 1 Department of Biomedical Sciences for Health, University of Milan, Luigi Mangiagalli 31, 20133 Milan, Italy; enrica.torretta@unimi.it (E.T.); pietro.barbacini@unimi.it (P.B.); 2 Ph.D. school in Molecular and Translational Medicine, University of Milan, 20142 Milan, Italy; 3 Chair for Biomarkers of Chronic Diseases, Biochemistry Department,College of Science, King Saud University, Riyadh 11451, Saudi Arabia; ndaghri@ksu.edu.sa; 4 I.R.C.C.S Orthopedic Institute Galeazzi, R. Galeazzi 4, 20161 Milan, Italy

**Keywords:** obesity, sphingolipid, osteoarthritis, cardiovascular disease, type 2 diabetes, gender, aging, hypoxia

## Abstract

This paper reviews our present knowledge on the contribution of ceramide (Cer), sphingomyelin (SM), dihydroceramide (DhCer) and sphingosine-1-phosphate (S1P) in obesity and related co-morbidities. Specifically, in this paper, we address the role of acyl chain composition in bodily fluids for monitoring obesity in males and females, in aging persons and in situations of environmental hypoxia adaptation. After a brief introduction on sphingolipid synthesis and compartmentalization, the node of detection methods has been critically revised as the node of the use of animal models. The latter do not recapitulate the human condition, making it difficult to compare levels of sphingolipids found in animal tissues and human bodily fluids, and thus, to find definitive conclusions. In human subjects, the search for putative biomarkers has to be performed on easily accessible material, such as serum. The serum “sphingolipidome” profile indicates that attention should be focused on specific acyl chains associated with obesity, per se, since total Cer and SM levels coupled with dyslipidemia and vitamin D deficiency can be confounding factors. Furthermore, exposure to hypoxia indicates a relationship between dyslipidemia, obesity, oxygen level and aerobic/anaerobic metabolism, thus, opening new research avenues in the role of sphingolipids.

## 1. Introduction

Sphingolipids (SLs) are molecular components of membranes responsible for their homeostasis, thus, changes in lipid composition affect not only membrane structure, but also receptor organization and function. Ceramide (Cer), sphingosine (Sph), and sphingosine-1-phosphate (S1P) are known to act as signaling molecules and are involved in the regulation of cell growth, differentiation, senescence, and apoptosis [1,2,3,4]. This review will provide a short overview of metabolism, localization, and compartmentalization of SLs in the frame of obesity in human subjects and animal models, highlighting similarities and discrepancies with the aim of defining which can be considered a possible biomarker that can be utilized in the clinical setting for obesity, and obesity-related co-morbidities as cardiovascular disorders (CVD), type 2 diabetes mellitus (T2DM) and osteoarthritis (OA) [5,6,7,8]. It is well established that high fat mass increases the risk of CVD and that waist circumference is a significant predictor of this syndrome [9,10]. Weight gain plays a central role also in the T2DM onset. The increase of non-esterified fatty acids, hormones, cytokines and pro-inflammatory markers is directly linked to insulin resistance, in which elevated plasma fatty acids cause a decreased glucose transport in muscle cells, as well as increased fat breakdown, leading to elevated hepatic glucose production. These events, together with pancreatic β-cell dysfunction, can lead to T2DM [11] in obese subjects, particularly in those unable to counteract insulin resistance [12].

Another recurrent co-morbidity of obesity is OA which affect both weight and not-weight bearing joints [13]. It has been described that high serum cholesterol levels are associated with OA severity and that lipids in obese subjects accumulate in chondrocytes [14,15]. However, a clear picture of the signaling network generated by lipids in obesity-associated OA is still missing.

### 1.1. Sphingolipid Synthesis

The SL metabolic pathway is an intricate structure that envelopes simple molecules like Cer and a multitude of complex glycosphingolipids, in which Cer represents the molecular core of biosynthesis and catabolism of SLs. Ceramide levels in cells can be associated with de novo synthesis and/or hydrolysis of complex SLs resulting from the degradation of glycosphingolipids (GSLs) or from hydrolysis of sphingomyelin (SM) [16,17]. In the de novo pathway, serine and palmitoyl-CoA are condensed by serine palmitoyltransferase (SPT) to produce 3-ketodihydrosphingosine, which in turn is reduced to dihydrosphingosine (sphinganine) [18]. Cer synthases (CerS1-6), *N*-acylate the sphinganine to produce dihydroceramide (dhCer) that undergo desaturation by dihydroceramide desaturase (DEGS) to finally generate Cer. By this pathway, several Cers with different acyl chain lengths can be produced, depending on the different CerS isoforms, from 1 to 6, that have a specific preference for a different chain length of fatty acyl-CoAs [19]. The biological role of products from different Cer isoforms generating Cers with different acyl length remain so far unexplained. The produced Cer can undergo three different pathways: It can be phosphorylated to Ceramide-1-Phosphate (C1P) [20]; converted to glycosphingolipid [21,22]; or used to synthesize SM [23]. Cer is also obtained from the breakdown of complex glycosphingolipids through the sequential removal of the hydrophilic portions of GSLs by specific hydrolases producing galactosylceramides (GalCer) or glucosylceramides (GlcCer), which in turn can be hydrolyzed by specific galactosidase or glucosidases to produce ceramide [24]. At variance, the SM hydrolysis is controlled by five main types of sphingomyelinase (SMase): Lysosomal acid Smase (ASMase); neutral Smase (Mg^2+^ dependent -NSMase- or Mg^2+^ independent); alkaline Smase or zinc-dependent acid Smase with different subcellular localization and pH. Their action on SM determines the release of Cer and phosphocholine [25]. Once released, Cer can undergo degradation as a result of acid, alkaline and neutral alkaline ceramidases (Cdases) regulated by pH and subcellular localization. Those enzymes remove the fatty acid (FA) from Cer producing free sphingosine (Sph). The latter can return to the SL pathway or be phosphorylated by the action of specific kinases (sphingosine kinases 1–2) to sphingosine-1-phosphate (S1P) [26]. S1P can be dephosphorylated to Sph by specific S1P phosphatases or can be metabolized by S1P lyase, resulting in the release of ethanolamine phosphate and hexadecenal [27].

**Figure 1:**
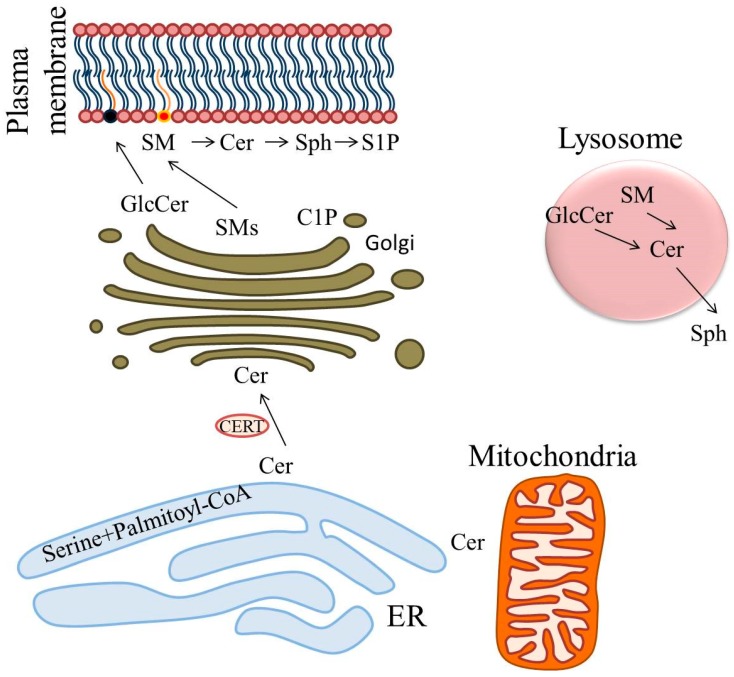
Sphingolipids biosynthesis and cellular trafficking. Endoplasmic reticulum (ER), ceramide (Cer), Ceramide transfer protein (CERT), sphingomyelins (SMs), Ceramide-1-Phosphate (C1P), glucosylceramides (GlcCer), sphingosine (Sph), sphingosine-1-phosphate (S1P).

The precise compartmentalization of these molecules regulates SL synthesis: The initial step occurs at the cytosolic side of the endoplasmic reticulum (ER), but also on ER perinuclear membranes or mitochondrial associated membranes (Figure 1). The second and third step occurs in the Golgi, in which more complex molecules are synthesized and transported to the plasma membrane through vesicular trafficking. From the plasma membrane, SLs can be recirculated through the endosomal pathway. SM and GlcCer are metabolized to Cer in the lysosomal compartment by Smases and glucosidases, and Cer are degraded by acid Cdase to form Sph. The latter, thanks to its positive charge, can leave the lysosome and move in the cytosol between membranes becoming available for recycling. This precise compartmentalization reinforces the concept that those molecules are not merely structural components, but actively participate to biological processes [21,22,23] and their dysregulation can have an impact on not only membrane structure dynamics, but also on signaling pathways involved in metabolic diseases.

### 1.2. Sphingolipid Analysis

Sphingolipids have been traditionally analysed by thin layer chromatography (TLC), which allows the separation and characterization of SLs by retention factors (Rfs) comparison with known standards [28]. More recently, SLs-specific ELISA immunoassays have been developed [29]; however, this methodology determines the SLs total content only, leaving undetected the saturated and unsaturated acyl chains. Furthermore, the wide dynamic range affecting sphingolipid composition in tissues and biological fluids requires sensitive technologies able to detect high and low abundant species. This goal was achieved with mass spectrometry, where different ionization methods [30,31] and analyzers [32,33,34] were used successfully. Furthermore, the high homology among similar sphingolipids and the consequent mismatch [35] can be overcome by high performance liquid chromatography (HPLC) [36], gas chromatography [37] and TLC-HPTLC [38,39,40], prior to mass spectrometry analyses.

Today, LC-ESI MS/MS is the most utilized method [41,42], but other on-line hyphenated techniques have been applied to sphingolipid research, like LC-NMR [43], CE-MS [44] and TLC/HPTLC-MS [45,46,47,48].

Despite cutting-edge technologies, there are still some concerns about sphingolipid analysis connected to SLs profiling in a single high-throughput analytical step. The latest generation mass spectrometers allow to get better inside into the sphingolipidome; however, the dynamic range is still far from being resolved and the complexity is due to the number of species and chemical structures that make their separation and quantitation extremely complex.

## 2. Sphingolipids and Obesity

The neologism “Globesity” perfectly defines the escalation of the pandemic of obesity, a complex status in which the increase in nutrient intake, adipocyte dysfunction, insulin resistance, ectopic fat accumulation and inflammatory status act in synergy. Obese subjects are characterized by inflammation associated with pro-inflammatory cytokines [49], availability of free fatty acids (FFA) [50] and oxidative stress [51]; all these features are connected with co-morbidities associated with obesity. The profound alteration of systemic metabolism in obesity and in obesity-related co-morbidities, severely affects SL homeostasis generating unbalance targeting adipose tissue, liver, skeletal muscle and cartilage [52,53,54,55]. In physiological conditions, FFA are used as an energy source; nevertheless, their increase leads to apoptosis, insulin resistance and to a number of alterations referred to as “lipotoxicity” [56]. In obese subjects, sphingolipid metabolism is affected by an increased bio-availability of FFA, which induce an increase of palmitoyl-CoA and a resulting increase of ceramide production via de novo pathway [57]. Studies on rodents show that saturated fatty acids can also stimulate the toll-like receptor 4 (TLR-4), activating Smases converting SM to Cer, thus, contributing to Cer levels [58,59]. Furthermore, the increase of pro-inflammatory cytokines, like IL-6 and TNF-α, observed in obese subjects can play a central in SL modulation. In C57BL/6J mice, the intraperitoneal administration of TNF-α induces upregulation of acid SMase, neutral SMase and SPT, suggesting that Cer levels can be increased not only through de novo synthesis associated with higher availability of palmitoyl-CoA precursors, but also to the indirect activity of toll-like receptor 4 (TLR-4) and proinflammatory cytokines [60].

It is well known that obesity is connected to inflammatory response, however, the precise mechanisms that trigger this response are not completely clear; nonetheless activation of TLR-4 by lipopolysaccharides (LPS) [61] or by FFA [62], macrophage infiltration [63], adipose tissue expansion [63] and hypoxia [64] have been proposed as causative effects, leading to the release of several pro-inflammatory cytokines, also known as adipokines [65,66]. The suggestion that saturated fatty acids could be potential activators of TLR-4 [62] links the Cer increase to inflammation [58]. By using in vivo and in vitro models, William L. Holland [58] demonstrated that saturated fatty acids lead to ceramide biosynthesis by activating TLR-4 and that increased Cer is required for TLR-4 dependent insulin resistance. TLR-4, is known to be responsible for initiating signals that activate NF-κB, JNK, and SOCS pathways [67,68] all factors involved in the regulation of inflammation. In 1992, it was demonstrated by KA Dressler et al. [69] that the increase of TNF-α was able to reduce the membrane sphingomyelin levels, increasing ceramide and suggesting that SMase was activated by TNF-α. This idea was further confirmed in 1994 by Wiegmann K et al. [70], which showed an independent activation of ASMase and NSMase by TNF-α, resulting in an increased ceramide production and activation of NF-Kβ by ASMase. In 2001, Terue et al. [71], demonstrated increased levels of Cer in brown adipocytes treated with TNF-α and glucose and that the relocation of GLUT-4 to the plasma membrane was inhibited by treating the same cell with a short-chain ceramide analogue (C2-ceramide). Furthermore, C2-Ceramide was suggested to be an activator of protein phosphatase leading to AKT dephosphorylation. Kolak et al. [72], confirmed that increased levels of ceramide and increased expression of SMases (SMPD1 and 2) characterized the subcutaneous adipose tissue of obese women with high levels of fat in the liver. Nevertheless, TNF-α increase was not statistically changed, whereas other markers of inflammation (i.e., macrophage marker CD68, chemokines monocyte chemoattractant protein-1 and macrophage inflammatory protein-1alpha) were found at variance in the group of patients with high fat liver, supporting the hypothesis that in obesity an increased inflammatory status impacts directly on ceramide level.

In ob/ob and db/db mice, transforming growth factor (TGF)-β, both mRNA and protein, were increased in adipose tissue [73,74]. Lin et al. described the role of TGF-β/Smad3 pathway in the regulation of insulin gene transcription and β-cell function [75]. Furthermore, it has been observed that a systemic blockade of TGF-β1 signaling protects mice from obesity, diabetes and hepatic steatosis [76]. In humans, TGF-β levels correlate with obesity [77,78].

Another point to be highlighted is the expression of the anti-inflammatory cytokines, like IL-10, which attenuates the inflammatory processes induced by TNF-α, the mode of action is still unknown, however [79,80]. Low levels of IL-10 are correlated with metabolic syndrome and T2D, whereas higher levels are detectable after weight loss [81].

In the context of SLs and obesity, TNF alpha/IL 10 balance counteracts the up-regulation of acid SMase, neutral SMase and SPT and the increment of Cer. In addition, IL-10, together with IL-13 inhibits the proinflammatory cytokine-induced degradation of SM to Cer through the activation of phosphatidylinositol 3-kinase [82]. The expression of other anti-inflammatory cytokines like adiponectin and leptin [83] allow to control the fat mass growth. These adipokines have beneficial effects on body weight regulating food intake/energy expenditure and reducing inflammation [84,85,86,87]). Rats treated with leptin display a blunted de novo ceramide synthesis and systemic improvement of insulin sensitivity [88]. Hypothalamic infusion of leptin in mice results in a reduction of total ceramide content in white adipose tissue (WAT) [89] through the repression of serine palmytoil trasferase. Adiponectin also exerts its beneficial metabolic effect by lowering cellular ceramide level. Furthermore, it is a potent anti-apoptotic in the context of cardiomyocytes and pancreatic β cells in vivo stimulating the production of sphingosine-1-phosphate through the involvement of its receptors, AdipoR1 and AdipoR2, that exhibit ceramidase activity in an adiponectin-dependent manner [90]. It is still unknown whether the receptors have ceramidase activity, or they act by sequestering or inducing ceramidase upon activation [91].

Concerning OA, obesity affects the chondrocytes responsiveness to leptin in OA patients [92]. Leptin may have a beneficial effect on cartilage synthesis, but an excess of leptin may decrease levels of extracellular matrix proteins leading to cartilage loss [93,94]. Since gangliosides are the most abundant glycosphingolipids in cartilage and contribute to condrocytes homeostasis [95] and differentiation [96], mice lacking GM3 synthase (GM3S; GM3−/− mice), are ganglioside deficient. The depletion of gangliosides enhances articular cartilage repair and regulate chondrocyte hypertrophy [55], suggesting these molecules as negative regulators of cartilage homeostasis.

Despite increasing evidence supporting the role of ceramides in obesity and in associated disorders, a clear portrait for different ceramide sub-species and their biological role is still lacking, making the characterization of their actions in obesity and related-comorbidities warranted.

### 2.1. Ceramide and Insulin Resistance

Ceramide represents the central node of SLs; it is the keystone of sphingolipid molecules, and it is localized in the middle of the sphingolipid pathway. Although it is not the most abundant SL in humans (accounting for 3% of the total SLs in serum) [97], it is the most studied molecule. In the cell, Cer exerts several actions [98] ranging from the induction of apoptosis [99], insulin resistance [100] and alterations of vascular tone [6]. The first study linking obesity, ceramides and insulin resistance was conducted in 1990 by Turinsky et al. [101] on Zucker diabetic fa/fa rats. In lean animals, 1,2-Diacylglycerol and ceramide levels were lower in muscle than in the liver, whereas in Zucker diabetic fa/fa rat livers and muscle ceramide levels were high and comparable, suggesting a connection between ceramide and insulin resistance. In vivo, the high-fat diet (HFD) or in vitro FFA administration has been widely adopted as tools to stimulate weight gain on animal and cell models to mimic fat accumulation that precedes obesity [102,103,104]. Such types of induced-obesity allow us to decipher the role of molecules without other confounding factors. Cell lines from murine myotubes [105] treated with radioactive palmitate or human female myotubes supplemented with palmitate [106], show increased palmitate incorporation into ceramide and increased ceramide production, further supporting that exogenous (dietary) oversupply of fatty acids, and specifically palmitate, is utilized to produce ceramide and can also contribute to increasing ceramide levels. Moreover, attention was focused on the acyl chain length of different ceramide species rather than total Cer, in order to associate specific acyl chain activities to chain length. Murine models, fed with HFD or treated with palmitate, have shown increased ceramide C16:0 and C18:0 levels independently from tissue or cell type [52,53,107,108,109,110,111,112]. However, discrepancies after HFD treatment were observed in the number of long-chain Cers in the liver, skeletal muscle, and adipose tissue (Table 1). Nevertheless, quantitative results have been contradictory: Cer C24:0 has been shown to decrease in the liver as described by Cinar and Turner [52,107] and increase as demonstrated by Bruce et al. [53], as well as C24:1, that was decreased in the study from Turner [52], but increased in other similar studies [53,108,113]. C24:1 gave conflicting results also in skeletal muscle, resulting from decreased as described by Bruce in 2013 [54], but increased in another study of 2012 from Bruce [53]. Conversely, Cer C22:0 decreased in adipose tissue according to Turpin [114], but increased in the study from Turner [52]. Total Cer increased in a study from Lyn-Cook et al. [115], which also demonstrated also that HFD increased the expression of Ceramide synthase, SPT and SMase in the liver. Ceramide was measured by dot blot in the liver and by ELISA in serum and results showed it increased in HFD-fed rats compared to LFD fed animals [116]. These results suggest that a careful evaluation of the efficiency of methods adopted for quantitation have to be considered. Ussher et al. [117] determined Cer levels derivatizing the molecule to o-phthalaldehyde to generate a fluorescent compound that was separated by HPLC and quantified by fluorescence spectrometry. In gastrocnemius, ceramide was doubled in HFD-fed animals, whereas hepatic ceramide levels were unaffected, demonstrating a countertrend to the Lyn-Cook data [115], which utilized a different fluorescent-based assay on the same animal model. Other discrepancies were described for sphingosine using LC-MS, in which sphingosine increased [118,119]; whereas when using fluorescence, no changes were detected [120] (Table 1), thus, confirming the relevance of methodologies in the quantitative assessment of these molecules. Levels of ceramide, insulin resistance and glucose intolerance observed in HFD mice were completely reversed by treatment with myriocin (a de novo Cer biosynthesis inhibitor). Utilizing C57BL/6J mice, Boini KM et al. [121] discovered a significant increase in plasma ceramide levels in HFD-fed mice and the treatment with amitriptyline (an inhibitor of both SMases and CDases) enabled the decrease of plasma ceramide levels. By studying non-alcoholic fatty liver disease (NAFLD) on Long-Evan rats, Longato L et al. [116] confirmed previous results highlighting the detrimental role of the HFD. In particular, compared to a low-fat diet (LFD), HFD led to NALFD, peripheral insulin resistance, increased expression of the pro-ceramides gene in the liver and increased levels of serum ceramides. Kurek et al. [120] confirmed that HFD by itself was able to determine NAFLD and induce insulin resistance in Wistar rats; nevertheless, treatment with myriocin was able to reduce DAG, TAG and Cer levels in the liver, leading to a reduction on NAFLD severity.

The use of leptin deficient ob/ob mice models partially confirmed these data, even if Samad et al. [60] detected a decrease of total Cer in adipose tissue, whereas other studies investigating the same HFD-fed animals indicated an increase of most of the acyl chain Cers in the same tissue [52,109,114] (Table 2). In human subjects, Haus JM et al. [122] found that obese T2DM patients had higher plasma concentration of total C18:0, C20:0, C24:0 and C24:1 Cer compared to controls and the same Cer chains were inversely correlated to insulin sensitivity. However, the same Cer C18:0, C20:0 and C24:1 were not detected as changed in serum of overweight adolescents by Majumdar [123] (Table 3). Concerning the quantitative assessment of different acyl Cer chains, a number of discrepancies in the data characterized both animal models and human subjects, thus, data have to be considered cautiously. Turpin SM et al. [114] demonstrated that CerS6 mRNA expression and Cer C14:0, C16:0, C16:1, C18:0, C18:1 and C22:1 were elevated in obese adipose tissue and that an increase in CerS6 positively correlated with insulin resistance in humans. These data were further confirmed by CerS6 deficient mice which were protected from HFD and showed reduced obesity, glucose intolerance and low levels of Cer C16:0. Recently, Raichur S et al. [124] used antisense oligonucleotide (ASO) in leptin deficient ob/ob and HFD mice models to demonstrate that CerS6 ASO treatment determined a reduction in the CerS6 expression in the liver and a decrease of subcutaneous and visceral fat. Furthermore, this treatment induced a reduction of C16:0 ceramides plasma levels, resulting in improved glucose tolerance and insulin sensitivity, confirming the strict correlation between ceramide, obesity and insulin resistance again. A recent study by Turpin SM et al. [114] revealed increased levels of C18:0 ceramide and Cers1 over expression in skeletal muscle of HFD-fed mice and in mice lacking Cers1, while a reduction in levels of Cer C18:0 was observed and was associated with improvement of glucose homeostasis. However, the muscle-specific deficiency of CerS5/6 failed to protect mice from obesity-induced insulin resistance [114], highlighting the relationship among acyl chain length and isoforms of Cers (1–6) enzymes. Data discrepancies are summarized in Table 1 and Table 2.

### 2.2. Role of Complex Sphingolipids

#### 2.2.1. Dihydroceramides

Dihydroceramides (Dhcers) have been long considered inactive precursors of ceramide generation during the de novo synthesis, since the short chain (C2-C6) ceramides nor their saturated counterpart was able to stop cell proliferation and induce apoptosis [127,128,129]. Only in 2006, a bioactive role for dhCer was first acknowledged by Zheng et al. [130], during a lipidomic screening of cultured cells treated with fenretinide, an anticancer drug supposed to induce cell death through ceramide-mediated action. After treatment, dhCer levels increased, whereas the expected increase of ceramide was not observed [130]. In 2013, Brozizick et al. [131] revealed that monkeys under a long period of HFD showed increased plasma levels of Cer and dhCer exacerbated when animals developed diabetes. In 2013, Lopez et al. [132] discovered that, among dhCers, only C24:1 were increased in plasma of obese female children and adolescents with T2DM. When performing the human lipidomic profile, Mamtani et al. [133] discovered a peculiar association between plasma levels of dhCers 18:0, 20:0, 22:0 and 24:1 and waist circumference in Mexican Americans. Bergman et al. [134] investigated the effects of exercise in relation to sphingolipid metabolism in human subjects, linked to dhCer and insulin resistance, and found that the total dhCer, together with C16:0 ceramide and C18:0 sphingomyelin, was higher in T2DM subjects and positively correlated with insulin resistance. More recently, a study by Wigger et al. [135] identified a specific group of sphingolipids that correlate with glucose intolerance and insulin secretion in mice (Table 4). Notably, dhCer C22:0 could be used in humans as biomarkers of T2DM disease progression. Several studies have described correlations among insulin and obesity, metabolic parameters and dhCers linking them to the development of metabolic disorders, though the pathophysiological mechanisms associated with these molecules are still unknown [136]. Most of the studies in the context of alteration rely on Des1, the enzyme that introduces the double bond in Cer producing dhCer. In one study, Mice Des1−/− resulted in incomplete penetrance lethality phenotype characterized by increased levels of dhCer and decreased levels of Cer [137]. Another study [138] described the increment of arterial ceramide-induced by HFD in Des1+/+, but not in Des1+/− mice, though Des1+/− HFD animals developed a severe increase of fat mass, decreased lean mass and impaired glucose tolerance than Des1+/+ mice. Nevertheless, in the study from Zhang, Q.-J et al. [138]. Des1+/- mice developed a more severe metabolic alteration compared to Des1+/− mice described by Holland et al. [137]. Both studies pointed out that the causative role for the metabolic alterations leading to insulin resistance and vascular dysfunction has to be ascribed to the rate of conversion of dhCer to Cer more than to dhCer itself. Conversely, Barbarroja et al. [139], indicated that both in vivo and in vitro Des1 reduction, impairs adipogenesis and lipogenesis, and that adipocyte treatment with dhCers resulted in metabolic dysregulation.

It can be concluded that Des1 is a key enzyme in sphingolipid generation and its activation or impairment is crucial for sphingolipids homeostasis. Nevertheless, it is still not completely clear if its role in metabolic alterations is due to its increase or due to its decrease. Further investigation is required to precisely define the pathophysiological mechanisms regulated by this enzyme, which are pivotal in obesity.

#### 2.2.2. Sphingomyelin and Obesity

Sphingomyelin is one of the most abundant sphingolipids in bodily fluids and in tissues, accounting for 87% of total plasma sphingolipids [97]. Correlations have been found among high levels of SM and cholesterol, coronary artery disease in humans and HFD and insulin sensitivity in mice [52,140,141]. Conversely, reduced levels of SM and increased SMases (both neutral and acidic) mRNA expression were observed in the adipose tissue of ob/ob mice, and the more significant decrease was observed in long chain SMs (20:0, 20:1, 22:1, 24:0, 24:1) [52,60] (Table 2). Interestingly, serum levels of SM increased in ob/ob mice, suggesting an intriguing SL flux from tissue to body fluids [60]. Utilizing C57BL/6J mice fed with HFD for ten weeks, Norris et al. found that the mice fed with HFD and supplemented with SM displayed reduced hepatic steatosis, lower cholesterol, lower liver TG accumulation and tissue macrophage infiltration, suggesting that SM dietary supplementation can ameliorate metabolic complications induced by HFD [142]. Localization of SM plays an important role in overweight and obesity. Hints on this direction were provided by Mitsutake et al. investigating the sphingomyelin synthase 2 (SMS2) KO mice [143]. These mice exhibited a reduced number of large and mature lipid droplets in the liver, caused by a lack of SM and by the association of SMS2 with the fatty acid transporter CD36/FAT and caveolin 1. In addition, SMS2 KO mice did not increase their body weight by HFD, suggesting SMS2 as a target to prevent high-fat diet-induced obesity and insulin resistance.

Studies on human subjects by Hanamatsu et al. demonstrated that in the obese, SM saturated acyl chains (C18:0, 20:0, 22:0, and 24:0) were increased compared to the control group and positively correlated with serum biochemical (TC, TG, and LDL-C) and insulin resistance parameters (HOMA-IR, AST and ALT) [144]. Recent metabolomic studies identified correlations between lysophosphatidylcholine (LPC) (lysoPC C17:0, 18:1, and 18:2) and obesity, whereas sphingomyelins (C18:1) and dhCers were associated with a pre-diabetic insulin state [145]. Bagheri et al., at variance, found a negative association between LPC and obesity (C18:1 and C18:2) in obese Iranian adults, without any correlation among SM and obesity [146]. On the other hand, by investigating metabolomics profiles and insulin resistance in European children, Hellmuth et al. [147] found that SM C32:2 was strongly associated with BMI z-score, whereas the association with HDL and body weight was absent. Recently, Im, et al. [148] found that SM concentration correlated positively with cholesteryl esters and waist-to-hip ratio and that sphingomyelin levels were higher in pre-diabetic men with abdominal obesity. The effects of SM in obesity or the obesity-induced generation of SM were less considered compared to Cer, even though their levels in obesity or HFD increased in plasma [60], liver [149], adipose tissue [52] erythrocyte and heart [149]. Altogether, these studies suggest that sphingomyelin and its different acyl chains can play a causative effect in the onset and development of obesity; however, it is important to note that the results are to some extent contradictory. In order to define the precise role of SM in obesity, strict control of sampling, the use of defined standards and the identification of standardized analytical procedure is required.

#### 2.2.3. Sphingosine-1-Phosphate (S1P)

Sphingosine (Sph) is produced by ceramide breakdown, and sphingosine-1-phosphate (S1P) represents its phosphorylated counterpart. Both molecules are key signaling bioactive elements in the liver [150], muscle [151], blood [152], and adipose tissue [153]. Despite their consistent signaling, studies investigating their role in obesity are limited compared to other SLs. Kowalsky et al. [126] investigated S1P plasma levels by a commercially available ELISA and their results indicated an increase of S1P in mice characterized by genetic and HFD-induced obesity. The study was extended to humans, and it identified a positive correlation among S1P and BMI, total body fat percentage and HOMA-IR. Notably, S1P levels increased in mice after 12h fasting, suggesting that the S1P pool is quickly modified and S1P can exert potentially positive effects, which contribute to recovery/survival of pancreatic β-cells during the compensatory hyperinsulinemia status in insulin-resistant and obese subjects. Revising the literature, studies on human subjects, as well as on animal models, provided different results even when using the same quantitative methodology. Majumdar et al. described no differences in S1P levels in obese adolescents [123], whereas Ito et al. [125] confirmed an S1P increase in the obese, in accordance with the results of Kowalsky (Table 3). The S1P increase in mice was confirmed by Kleuser group [154], which identified increased levels of hepatic S1P in HFD-fed New Zealand obese mice. An increase in S1P was also seen in the primary culture of rat and human hepatocytes, supplemented with palmitate. Furthermore, the S1P increase induced a decreased insulin stimulation mediated by the sphingosine-1-phosphate receptor 2, suggesting a regulatory effect of S1P on insulin signaling in hepatocytes. These observations were not confirmed by Kurek et al. [120]. In a recent paper, the group of Kleuser [155] was able to clarify in vivo the apparent discrepancy between the pro-survival effect of pancreatic β-cell suggested by Kowalsky and the increased hepatocyte insulin resistance mediated by S1P. When analyzing New Zealand obese mice (which develop β-cell loss under HFD), Kleuser et al. identified an increase in T2D prevalence, paralleled by increased levels of S1P in plasma. Mice were treated with specific sphingosine-1-phosphate receptor 2 antagonist (JTE-013) avoiding disruption of insulin signaling and β-cell dysfunction, suggesting that S1P can act on pancreatic β-cell by increasing its levels or by binding to different receptors; thus, it can lead to a negative or positive outcome. In 2012, Moon et al. [156] identified in C57B/6J mice and in 3T3-L1 cells, the central role of the sphingosine 1 phosphate analog (FTY720) in the regulation of adipogenesis and lipolysis. This molecule was able to down-regulate adipogenesis and enhance lipolysis, both in vivo and in vitro, introducing a new avenue in the treatment of obesity. Interestingly, the same group [157] demonstrated that S1P was able to reduce TG accumulation and inhibit differentiation in 3T3-L1 preadipocytes, resulting in the downregulation of markers of adipogenic differentiation, like peroxisome proliferator activated receptor γ (PPAR γ) and CCAAT/enhancer binding protein and adiponectin. Surprisingly, it has been reported that the action of S1P and FTY720 toward the S1P receptor 1 is different, as it is based on the recycling of the ligand in the former and degradation in the latter [158]. A positive role for S1P in obesity was also observed by Silva et al. [159] which, in 2014, identified the S1P/S1P receptor1 axis as a central node in energy homeostasis. S1P intracerebroventricular injection was able to reduce food intake, increasing rat energy expenditure, highlighting questions on the differences between exogenous and endogenous sphingolipids. Levels of S1P and sphingosine are finely tuned inside cells, tissue and in body fluids and are regulated by sphingosine kynases 1–2 (SphK1–2) and sphingosine phosphatases [27]. The administration of exogenous palmitate to C2C12 myotubes was able to increase levels of S1P and the activity of SphK1 [105]. Notably, this was not observed when myotubes were treated with oleate, suggesting that Sphk1 is activated only in the presence of palmitate. In 2013, the same group was able to dissect the mechanism through which palmitate was able to induce a SphK1 increase in muscle [160]. Sphk1 overexpression was linked to the interaction between peroxisome proliferator-activated receptor-α (PPAR-α) and SphK1 promoter. However, it remains unclear if SphK promoter induction was in response to direct PPAR-α interaction or due to PPAR-α downstream signaling. In 2018, Nagahashi et al. investigated the link among obesity, inflammation and cancer and confirmed that in breast cancer patients and animal models the HFD, alone was able to induce the axis SphK1/S1P/S1PR1 and to stimulate in obesity a cancer-associated pro-inflammatory cytokines (IL-6 and TNF-α) [161]. Treatment with FTY720 was able to reduce activation of the S1P axis and levels of cytokines, resulting in prolonged survival of HFD-administered tumor-bearing mice. Not only SphK1, but also SphK2 contribute to the action of S1P. Notably, SphK2^−/−^ mice are protected from diet-induced obesity and develop a lean phenotype and improved glucose tolerance [162] and are characterized by a two-fold increase of S1P in plasma, suggesting that the inhibition of SphK2 contributes to the maintenance of high levels of S1P and may be potentially positive in the treatment of obesity. In view of these studies, it can be hypothesized that the S1P increase follows two possible mechanisms: The upregulation of SphK1, already seen in other animal models lacking SphK2 [163]; or the inhibition of the SphK2 gene [164,165].

**Figure 2:**
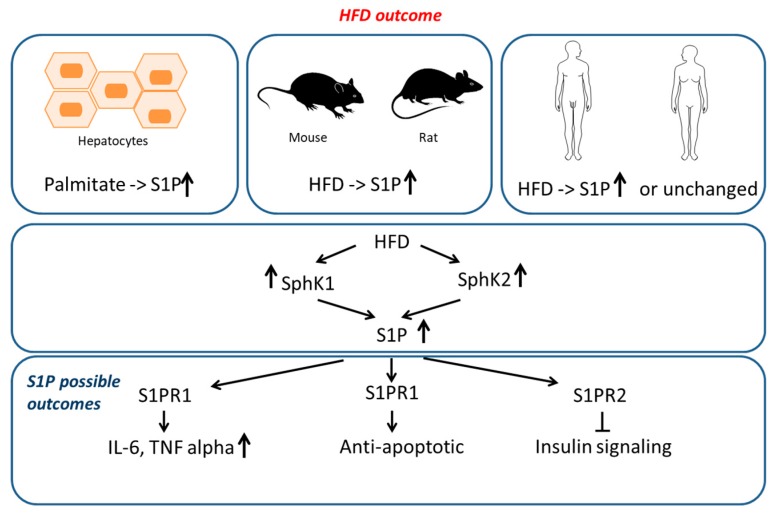
Sphingosine-1-phosphate (S1P) increases after palmitate supplementation (in cells) or after HFD (in animal models). In humans, the results are conflicting. Concerning S1P possible outcomes, the axis SphK1-S1P-S1PR1 (sphingosine kinase-1/ sphingosine-1-phosphate/ sphingosine-1-phosphate receptor 1) controls energy homeostasis, and an unbalance can increase the production of inflammatory cytokines. The S1P increase also induces a decreased insulin stimulation mediated by sphingosine-1-phosphate receptor 2 (S1PR2).

Collectively, this data indicates that S1P is an important player in obesity and obesity-related comorbidities, through its pleiotropic intracellular and extracellular activities targeting different pathways, even if its role remains so far unclear. The outcomes of the S1P increase is at variance in human and animal models, being protective in obese human subjects and detrimental animal models suggesting that in obesity animal models cannot recapitulate the human conditions (Figure 2).

## 3. The Influence of Gender

Sexual dimorphism in body composition and fat accumulation emerges during puberty, becoming evident in adulthood [166]. Men have greater muscle mass and reduced limb fat, but tend to accumulate abdominal fat. These visceral depots drain into the portal vein and the continuous exposure of the liver to free fatty acids and inflammatory cytokines, can result, via Randle’s effect [167], in the development of hepatic insulin resistance and type 2 diabetes (known as “Portal-visceral hypothesis” [168,169,170,171]). Conversely, women accumulate subcutaneous fat in the gluteofemoral area [172,173]. Interestingly, liposuction of thigh fat is followed by re-accumulation of fat in the abdominal area, suggesting a protective role of peripheral fat storage from the expansion of central fat depots [174].

Human adipocytes express sex steroid receptors [175,176]. Estrogens induce leptin secretion, whereas androgens inhibit it, more in adipocytes derived from women than men, acting in a sex-specific manner [177,178]. Total ceramides increase more in adipocytes of obese men than in women (34% vs. 31%, respectively) with an increase of Cer C18:1 in men and of Cer C24:1 in women [179]. Furthermore, in women, levels of ceramide C16:0 in adipocytes negatively correlates with levels of adiponectin in plasma, whereas total ceramides positively correlate with HOMA-IR. In men, ceramides containing saturated fatty acids, positively correlate with plasma IL-6 concentration.

In mice, ERα deficient females exhibited excess weight gain, increased visceral fat, glucose intolerance, insulin resistance, and dyslipidemia [180]. In ob/ob mice, urine and serum metabolic profiling by NMR spectroscopy highlight that insulin signaling, glycolysis and galactose metabolism were enriched in male mice only, whereas lipid metabolism, including sphingolipid metabolism and phospholipid biosynthesis was specific of female mice [181]. After the HFD diet, male mice’s brains had higher levels of saturated FAs and lower levels of polyunsaturated FA compared to females exposed to the same diet [182]. Interestingly, levels of palmitic acid (C16) was increased not only in HFD, but also in normal diet male mice compared to females. As a result, elevated levels of ceramides containing saturated fatty acids were detected in the hypothalamus of male mice, together with higher concentrations of sphingomyelins and glucosylceramide, which are precursors of gangliosides that block insulin receptor signaling. It has been demonstrated that pharmacological inhibition of glycosphingolipid synthesis ameliorates insulin sensitivity in ob/ob mice, HFD mice and in Zucker diabetic rats [183]. Recently, hepatic lipids were quantitated in 100 strains of mice and most lipids revealed large sex differences. In particular, Cer C16:0 and C18:0 were more abundant in females than in males, and even if Cer C16:0 correlated with insulin resistance and hepatic steatosis, females resulted less insulin-resistant than males and the lower levels in males can be explained by testosterone-mediated repression of ceramide synthase 6. Conversely, Cer C20:0 was higher in males and was associated with insulin resistance in males only.

However, the sex-specific role of sphingolipids in metabolic dysfunctions associated with obesity is far from being understood and studies addressing differences in sphingolipids levels in men and women are still in their infancy. Muscle ceramide was found to be similar between the two sexes in human subjects [184], whereas, in plasma, newborn and toddler children, sex-specific differences in sphingolipid concentrations were already evident, with a higher SM concentration in girls [185]. Higher levels of C18:0 and C18:1 SM, together with higher C1P C18 and dhCer C18 C22 and C24 levels, were also observed in the serum of healthy women compared to men [97], and also in Sprague-Dawley female rats [186]. A metabolomics profiling on 3000 subjects confirmed that higher levels of sphingomyelins were present in women [187]. In obesity, serum SM levels were higher in women, whereas SMs were lower in diabetic than non-diabetic patients [188]. The dependence of sphingomyelin levels on estrogen metabolism was recognized by Merrill A. et al. in 1985 [189]. Furthermore, higher levels of HDL in females [190] could explain, at least partially, higher sphingolipid levels.

Concerning ceramide, dihydroceramide, hexosylceramide, and GM3 levels, a study by Bui HH et al. in 2012 described high levels of these sphingolipids in women [191]. However, the very limited sample size (three women and five men) was not able to reveal sex-differences in the SLs levels.

In a cohort of more than 1000 Mexican Americans (39.1% men), ceramide C22:0, C24:0 and C24:1 were found to be significantly higher in the plasma of men than in women [192]. This increase was explained by increased activity of the enzyme ceramide synthase 2. Cer C18:0 and all dhCer were associated with BMI, suggesting an up regulation of the de novo synthesis. SM, as in previously mentioned studies, was lower in men and negatively associated with BMI.

Our study in men and women from Saudi Arabia (submitted to scientific report in 2019), partially confirms these data, with higher levels of long-chain Cers (C20:0, C24:1, C24:2) in normolipidemic normal weight men compared to women. However, also total SM, SM C18:0, C20:0, C22:1 and C24:1 were increased in men, whereas lactosylceramide (LacCer) C18:0 were increased in women. In dyslipidemic normal weight subjects, SM C22:0 was higher in women than in men, whereas in obese subjects, dhSM C18:0 levels were higher in men, and SM C14:1, total GlcCer and GlcCer C16:0 were higher in women. The increase of hexosylceramide in obese women can be related to the overexpression of hexosylceramide synthase in adipocytes that impairs insulin signaling [193]. Hexosylceramide has been described as a possible inducer of plaque inflammation and instability [194]. Dyslipidemic and obese women showed increased levels of dhS1P that can reduce dhCer, leading to the activation of the dhS1P pathway, and thus, reducing Cer generated from de novo synthesis.

The control exerted by sex-hormones on body fat distribution is confirmed in menopause, during which body shifts to an android-shape become evident [195,196,197]. Premenopausal women are protected from metabolic syndrome compared to age-matched men [198,199,200,201], suggesting the protective role of estrogens lost during menopause [202]. In addition, premenopausal women have a better lipid profile both in fasting and postprandially, with higher high-density lipoprotein (HDL)-cholesterol levels and lower low-density lipoprotein (LDL)-cholesterol and very low-density lipoprotein (VLDL)-cholesterol and triglyceride levels.

Estradiol is also able to stimulate sphingosine kinase-1 (SphK1) and the release of the protective factor S1P [203]. Abildgaard J. et al. [106] investigated muscle satellite cells isolated from vastus lateralis of premenopausal and postmenopausal women treated with palmitate during differentiation. Palmitate treatment induced a 108% increase of Cer in myotubes isolated from postmenopausal women compared with a 26% increase in myotubes from premenopausal women. Furthermore, mRNA expression of serine palmitoyltransferase1 (SPT1) after one day of palmitate treatment was significantly higher in postmenopausal myotubes compared with premenopausal, indicating that postmenopausal women are more likely to develop lipid accumulation and insulin resistance if chronically exposed to saturated fatty acid. The situation in biological fluids is more complex. In a paper published in 2015 [204], plasma concentrations of Cer and DhCer species were measured in men and postmenopausal women. Levels of Cer C16:0 were lower in women than in men in the 55–64-year-old group, but higher in women, the 65–74-year-old group. Conversely, Cer C24:0 was lower in women than in men over the age range of 55–94 years. Cer C26:0 increased with age in men, but decreased in women. Recently [205], a positive association has been described in women among age and plasma ceramide C24:0 and C24:1 levels, whereas in men, C24:1 was positively age-correlated. In women (both premenopausal and postmenopausal), Cer C24:1 was negatively correlated with plasma estradiol. DhCer C20:0 and C24:0 increased with age both in men and women.

The precise biological functions of these dimorphic changes need to be further investigated to understand if these data could have a biological explanation and can be a hint for personalized medicine. Moreover, in this case, there is a profound need for standardized methodologies and sampling to truly determine variation in this only apparently chemically simple class of molecules. At present, we can track this information.

## 4. The Influence of Age

Ageing is a physiological process characterized by decreased immune functions and stress response. Oxidative and metabolic stress may change the metabolism of sphingolipid, leading to increased incidence and risk for age-related diseases. In particular, recent studies have linked alterations in SM metabolism to different types of cancer and neurodegenerative disorders, caused by synaptic dysfunction [206,207]. A pivotal role is played by neutral sphingomyelinase (NSMase), the enzyme catalyzing the breakdown of SM to Cer that is extremely sensitive to oxidative stress caused by decreased hepatic glutathione levels, which are constitutively up-regulated in ageing [208].

This was described in rats by monitoring the activity of enzymes involved in sphingolipid metabolism in kidney, liver and brain tissues during development and ageing. The NSMase activity was increased in the liver, leading to ceramide overproduction. Interestingly, among the considered 21 enzymes, the activity of 18 of them was further increased in 24 months compared to six month- old rats [209]. In the brain, ceramide content also increased in ageing [207,210], and striatum and hippocampus membranes of ageing rats were enriched in NSMase [210].

Cutler and Mattson hypothesized that long chain sphingolipids (in particular, ceramide C18 and above) were responsible for ageing and age-related diseases [211]. They suggested that long-chain sphingolipids were unable to maintain the interbilayer movement, and this could hamper their passage across membranes, accumulating in subcellular regions [212]. In their model, sphingolipids at low concentration promoted cell survival and division; at higher levels promoted differentiation; and at highest levels induced apoptosis.

In human obesity, the increase of ceramide in response to the high-fat diet has been linked to ageing. In fact, ceramide accumulates more in the muscle of older subjects compared to younger ones [106,213,214,215]. In C57BL6 mice, after 12 weeks of HFD, the hearts of young mice did not show changes in CD36 protein expression, whereas in middle-aged mice a 1.5 fold increase was detected in HF-fed mice compared with the low fat-fed counterparts. Furthermore, middle-aged mice displayed a higher degree of cardiac hypertrophy compared with HFD young mice. Conversely, high-fat fed CD36 knockout mice were protected against cardiac hypertrophy. Myocardial ceramide levels were significantly increased in HFD middle-aged mice compared with LFD controls. These authors suggested an inhibition of AMP-activated protein kinase (AMPK), which behaves as a negative regulator of cardiac hypertrophy, activating the mTOR/p70S6K pathway, which increases protein synthesis associated with cardiac hypertrophy. In another study, old HFD-fed mice showed high levels of myocardial collagen, triglyceride, diacylglycerol, and ceramide, whereas pyruvate-dependent respiration and mitochondrial biogenesis was reduced in old mice, but also in young HFD mice [213]. Wistar old rats receiving an HFD were insulin-resistant and sarcopenic, showing a decreased muscle protein synthesis linking HFD not only to insulin resistance, but also to the loss of muscle mass and strength [215]. Furthermore, in these animals, intramuscular ceramide increased, while lipid storage capacity and ability to oxidize fatty acids were decreased.

In serum, already cited studies from Mielke et al. and from Vozella et al. highlighted the increasing levels of DhCer C20:0 and C24:0 in elderly subjects and the positive association between age and ceramide C24:1 [204,205].

The emerging data suggest that dietary, by caloric restriction, and pharmacological manipulations of SM metabolism, by NSM inhibitor, might be useful in extending lifespan and prevent obesity-related cardiomyopathies [216].

## 5. Can Environment Influence SLs Levels and Contribute to Obesity?

**Figure 3:**
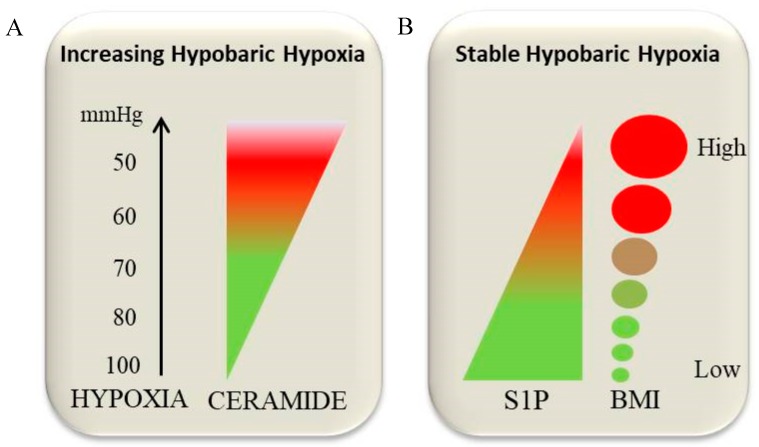
Influence of increasing hypobaric hypoxia (**A**) and of stable hypobaric hypoxia (**B**) on ceramide and S1P levels. Green color in (**A**) represents low levels of Cer while red represents high levels of Cer. Green color in (**B**) represents high levels of S1P and low BMIs, while red represents low levels of S1P and high BMIs.

Hypoxia is a phenomenon occurring in response to O_2_ restriction, usually caused by two conditions: An ischemic phenomenon or environmental exposure, like high altitude. In response to hypoxia, organisms undergo local/systemic responses and adapt to high altitude, whereas in ischemia the oxygen lack is caused by reduced blood flow (or reduced O_2_ in the blood) resulting in tissue hypoxia leading to necrosis. Previous reports have investigated the role of sphingolipids metabolism as a mediator of hypoxic stress [217,218] (Figure 3A).

Cer has been identified as one of the first sphingolipids acting during ischemia reperfusion (IR) mediated apoptosis. In 1997, Bielawska et al. [219] investigated cardiomyocytes in vivo and in vitro and found that C2-Cer, a cell-permeable Cer analog, was able to induce the apoptotic cell death of cardiomyocytes and ischemic myocardium with high levels of Cer. Reperfusion was able to reduce Cer levels in cardiomyocytes in vitro, but it was unable to restore basal levels of ceramides in vivo. In the same year, Cer involvement in ischemia reperfusion was again confirmed in the livers and kidneys of rats and mice, respectively [220,221].

In the last 20 years, not only Cer, but also SL metabolism have been identified as important players in hypoxia. In 2002, Yun and Kester [222] found out that S1P plays a role in ischemia-reperfusion (IR) and, specifically, that vascular smooth muscle during hypoxia showed increased levels of S1P, reduced levels of Cer and reduced activity of SMases. Ader et al. [223] highlighted the association of hypoxia and S1P on solid tumors, which were characterized by hypoxia, due to reduced vascularization. In this paper, SphK1 was stimulated and regulated both by low oxygen condition and by reactive oxygen species. This stimulation enabled the stabilization of the master regulator of hypoxia, the hypoxia inducible factor 1-α (HIF1-α), and its accumulation. Furthermore, the pharmacological inhibition of SphK1 hampered HIF1-α accumulation, leading to HIF1-α degradation, further confirming the modulation of HIF-1α by SphK1. The emerging role of SphK1 was also investigated by Schwalm et al. [224], which showed, in human endothelial cell line and in 926 subjected to hypoxia, that mRNA, protein expression and activity of SphK1 was increased, while SphK2 mRNA expression was unaffected. Furthermore, by depleting alternatively HIF-1/2α by siRNA, these authors demonstrated that gene transcription of SphK1 was controlled by HIF-1α and HIF-2α. It is now recognized that, in solid tumors, hypoxia activates HIF1-α and partially HIF2-α, which in turn activates the SphK1 transcription [218,225,226].

Following these results on “pathological” hypoxia, we focus our attention to the link between high altitude adaptation to hypoxia and the metabolism of sphingolipids. Systemic alterations can be observed in subjects permanently exposed to high altitude hypoxia, despite their adaptation, and these changes associated with their physiological adaptation can affect different populations inhabiting high-altitude lands [227,228,229,230,231,232].

Mice subjected to a 10% O_2_ hypoxic environment from birth develop classic signs of high-altitude adaptation: Polycythemia with nearly a two-fold increase in hematocrit levels (after eight weeks of hypoxia) and increased right ventricle mass compared to controls. Furthermore, after 10 days of hypoxia, cardioprotective molecules were substantially decreased, whereas pro-apoptotic molecules were increased [233]. In humans, hypoxia decreases proteins involved in iron transport, tricarboxylic acid (TCA) cycle, oxidative phosphorylation, and oxidative stress responses. HIF-1alpha and pyruvate dehydrogenase kinase 1 (PDK1) were still at the pre-hypoxia levels, whereas the mammalian target of rapamycin (mTOR), a marker of protein synthesis, was reduced [231,234,235,236]. Alterations of both ceramide and dhCer levels were observed during development in the right and left ventricle in control and hypoxic mice [237]. The hypoxic right ventricle, showed a significant increase in C16:0 dhCer levels, starting from week 1; this was accompanied by a significant decrease in the levels of its derivative C16:0 Cer. Not only did Noureddine et al. [237] detect alterations of dhCers during hypoxia, Devlin et al. [238] also identified in mice lung and in several mammalian cell lines a rapid, time dependent up-regulation of dhCer levels upon hypoxic insult in all cell lines, suggesting impaired dhCers desaturation by DEGS1/2, due to reduced O_2_ availability and oxidative stress [239,240].

S1P, due to its long-established role in hypoxia and IR, has been involved in the last 10 years studies, according to the idea that S1P can act as a therapeutic agent, mimicking the protection conferred by hypoxic pre-conditioning [241,242]. This has led to several pre-clinical investigations on its role.

Treatment of rats, prior to hypobaric induced hypoxia, with exogenous S1P ameliorated the acclimatization response through HIF1-α accumulation resulting in improvements of blood oxygen carrying potential, increased hemoglobin, hematocrit and RBC count, while control rats developed increased respiratory alkalosis and hypocapnia [243].

It is known [243,244,245,246] that one of the positive mediations of S1P on hypoxia-induced disturbances is obtained through the stabilization of HIF1-α; nevertheless, mechanistically a clear explanation is still required. In a 2016 work by Chawla et al. [247], rats treated with 1ug/kg b.w. S1P, prior to hypobaric hypoxia, showed HIF1-α accumulation mediated by S1P-induced activation of a small g-protein, called Rac1. Rac1 is an activator of HIF1-α [248,249]; nevertheless, its activation by S1P and the subsequent stabilization of HIF1-α were never reported before.

Recent work highlighted mechanisms of adaptation to high-altitude hypoxia in humans. In particular, the work focused on the capacity of red blood cells (RBC) to deliver O_2_ in relation to S1P levels. RBC are known to be not only a reservoir for S1P in circulation, but also producers of this molecule being SphK1 present in RBCs. However, the S1P degrading enzymes have never been detected in cytosol [250].

In Sun et al. [228], the authors detected S1P increased levels in blood paralleled by an increase of SphK1 and hemoglobin O_2_ release from day 1 to day 16 in human subjects exposed to altitude (5260 mt). By further assessing changes in S1P on mice, the authors were able to show that S1P was capable of inducing O_2_ release by anchoring to deoxygenated hemoglobin (Hb) and inducing deoxyHb binding to erythrocyte membrane, thus, facilitating the release of glycolytic enzymes. The release of glycolytic enzymes boosts glycolysis, which in turn permits the production of 2,3 biphosphoglycerate (2,3 BPG), which promotes O_2_ release from Hb, and thus, contributes to tissue oxygenation [229,230].

In this context, we have recently investigated the role of SLs in the adaptation to hypoxia in Andean children born and living at high altitude, characterized by different BMI and high levels of circulating lipids, despite similar daily activities and NFD diet. In this study, we identified differences in S1P levels between different body mass index categories (Figure 3B) [231]. In particular, normal weight (NW) and underweight (UW) children showed higher levels of S1P compared to obese (O), while ceramides were increased only in NW compared to both UW and O. It has been possible to speculate that UW and NW children adapted better to high altitude hypoxia living conditions, due to their constitutional high levels of S1P that permit the activation of RBC glycolysis leading to an improved release of O_2_ from red blood cells to tissues and reduced HIF1-α activation. The latter is known to be a lipogenetic inducer through direct PPARγ and FABP induction [232,233,251]. Conversely, obese children with reduced levels of S1P were unable to adapt to oxygen lack, due to their poor S1P-mediated O_2_ release, resulting in a constitutive activation of HIF1-α, causing an accumulation of lipids, due to both lipogenesis and ceramide-mediated lipotoxic effects [98,252,253]. This pioneering study on the relation between obesity and S1P levels in high altitude dwellers is currently under investigation in our lab in an attempt to associate the genetic background with metabolic adaptation to hypoxia, also considering a population which is well adapted to this difficult environmental condition, such as the Tibetans.

Regarding other sphingolipids and their roles in high altitude adaptations, Gong et al. [252] identified monosialotetrahexosylganglioside 1 (GM1) as a therapeutic molecule for treatment of high-altitude cerebral edema. Rats subjected to a treatment with GM1 (10 or 20 mg/kg b.w.) prior to high altitude exposure showed reduced brain vascular leakage, which was confirmed by higher occludin levels and lower aquaporin-4 expression. At the same time, GM1 administration reduced inflammation observed in rats by reducing IL-1β, TNF-α and IL-6 levels. Another investigation [253], conducted in human subjects which had developed high-altitude pulmonary edema (HAPE) after high altitude exposure, demonstrated that plasma C8-ceramide, sphingosine and glutamine could be used as diagnostic biomarkers for HAPE, suggesting that S1P levels could be used for monitoring adaptation to hypoxia. Finally, a work from 2019 by O’Brien et al. [254] on human subjects ascending to Mount Everest Base Camp, identified a decrease in TG alongside an increase in plasma levels of free fatty acids (palmitic, linoleic and oleic acids) and an increase in SM 34:2. Unfortunately, the work did not mention ceramide levels in subjects; this would have been of interest given the increased levels of circulating free fatty acids (in particular palmitate) that are known to be central in ceramide de-novo biosynthesis [57,105,106].

## 6. Conclusions

In conclusion, this class of molecules plays an important role in obesity and obesity-related co-morbidities. However, when considering circulating bodily fluids instead of changes in “total SL species” influenced by dyslipidemia, better insight the chemical composition of single species will be required in order to establish with sufficient accuracy which species can be selected as biomarker in obesity. Variations in SLs’ acyl chains characterized by obesity and dimorphic changes suggest that the focus has to be on specific Cer acyl chains, and this represents a new challenge to separation science and mass spectrometry approaches.

The study of high-altitude inhabitants indicated the same dependency of Cer and SM on dyslipidemia, further supporting the point that more work has to be done to identify the precise acyl chain composition of each single species. Furthermore, S1P appears to be a key regulator of hypoxia adaptation and hypoxic obesity, opening a new avenue in the field of hypoxia tolerance in high altitude inhabitants, but also in the relationship between obesity, oxygen levels and aerobic/anaerobic metabolism.

## Figures and Tables

**Table 1:** Discrepancies in alteration of sphingolipid species in high-fat diet (HFD) animal models.

Tissue	Sphingolipid Species	Increased/ Decreased	References	Experimental Model	Quantification Method
LIVER	Cer C24:0	Decreased	[52,107]	C57BL/6 mice HFD (60% fat) fed for 16 weeks	LC-MS/MS
				C57BL/6 mice HFD (42% fat) fed for 16 weeks	LC-MS/MS
		Increased	[53]	C57BL/6 mice HFD (42% fat) fed for 6 weeks	LC-MS/MS
	Cer C24:1	Decreased	[52]	C57BL/6 mice HFD (42% fat) fed for 16 weeks	LC-MS/MS
		Increased	[53,108,113]	C57BL/6 mice HFD (42% fat) fed for 6 weeks	LC-MS/MS
				Wistar rats HFD (34% fat) fed for 3 weeks	scraped off TLC+GC-LC
	Total Cer	Increased	[115,116]	C57BL/6 mice HFD (60% fat) fed for 20 weeks	Nile Red fluorescence-based assay
				Long Evans rats HFD (60% fat) fed for 8 weeks	Nile Red fluorescence-based assay
		No change	[117]	C57BL/6 mice HFD (60% fat) fed for 12 weeks	Derivatizion to o-Phthalaldehyde+LC-fluorescence
	Sphingosine	Increased	[118,119]	Syrian Golden hamsters HFD (30% fat with 40% fructose) fed for 2 weeks	LC-MS/MS
				C57BL/6 mice HFD (58% fat) fed for 16 weeks	LC-MS/MS
		No change	[120]	Wistar rats HFD (60% fat) fed for 5 weeks	scraped off TLC+GC-LC
					Derivatizion to o-Phthalaldehyde+LC-fluorescence
SKELETAL MUSCLE	Cer C24:1	Decreased	[54]	C57BL/6 mice HFD (42% fat) fed for 6 weeks	LC-MS/MS
		Increased	[53]	C57BL/6 mice HFD (42% fat) fed for 6 weeks	LC-MS/MS
ADIPOSE TISSUE	Cer C22:0	Decreased	[114]	C57BL/6 mice HFD (55.2% fat) fed for 14 weeks	LC-MS/MS
		Increased		C57BL/6 mice HFD (42% fat) fed for 16 weeks	LC-MS/MS

**Table 2:** Discrepancies in alteration of sphingolipid species between ob/ob mice and HFD fed animals.

Tissue	Sphingolipid Species	Animal Model	Increased/ Decreased	References	Experimental Model	Quantification Method
ADIPOSE TISSUE	Total ceramide					
	Cer C16:0, C18:0, C18:1, C20:0,	ob/ob mice	Decreased	[60]	C57BL-ob/ob mice	LC-MS/MS
	C22:0	HFD fed animals	Increased	[52,109,114]	C57BL/6 mice HFD (42% fat) fed for 16 weeks	LC-MS/MS
					C57BL/6 mice HFD (60% fat) fed for 16 weeks	LC-MS/MS
					C57BL/6 mice HFD (55.2% fat) fed for 14 weeks	LC-MS/MS
	Total SM					
	long chain SMs (20:0, 20:1, 22:1, 24:0, 24:1	ob/ob mice	Decreased	[60]	C57BL-ob/ob mice	LC-MS/MS
	SM 14:0, SM 16:0, SM 16:1, SM 18:0 and SM 18:1	HFD fed animals	Increased	[52]	C57BL/6 mice HFD (42% fat) fed for 16 weeks	LC-MS/MS

**Table 3:** Discrepancies of sphingolipid species between plasma and serum in obese human subjects. Type 2 Diabetes (T2D).

Biological Fluid	Sphingolipid Species	Increased/ Decreased	References	Subjects	Quantification Method
PLASMA	S1P	Increased	[125,126]	Obese adults	HPLC
				Obese adults	ELISA
SERUM	S1P	No change	[123]	Overweight adolescent	LC-MS/MS
PLASMA	Cer C18:0, C20:0, C24:1	Increased	[122]	Obese T2D adults	LC-MS/MS
SERUM	Cer C18:0, C20:0, C24:1	No change	[123]	Overweight adolescent	LC-MS/MS

**Table 4:** Dihydroceramides (DhCer) species changes in plasma and serum of type 2 diabetes mellitus (T2DM) human subjects. Waist circumference (WC).

Biological Fluid	DhCer Species	Increased/ Decreased	References	Subjects	Quantification Method
Plasma	DhCer 24:1	Increased	[132]	Obese female children and adolescents with T2DM	LC-MS
Plasma	DhCers C 18:0, 20:0, 22:0 and 24:1	Associated with WC	[133]	San Antonio Family Heart Study	LC-MS
Serum	Total dhCer	Increased	[134]	T2DM subjects	LC-MS
Plasma	DhCer C22:0	Increased	[135]	T2DM subjects and pre-diabetic mice	LC-MS

## Abbreviations

Cer	ceramide
SM	sphingomyelin
dhCer	dihydroceramide
S1P	sphingosine-1-phosphate
Sph	sphingosine
C1P	ceramide-1-phosphate
SLs	sphingolipids
GSLs	glycosphingolipids
CerS	ceramide synthase
DEGS	dihydroceramide desaturase
GalCer	galactosylceramides
GlcCer	glucosylceramides
SMase	sphingomyelinase (): lysosomal SMase (); SMase
ASMase	acid sphingomyelinase
NSMase	neutral sphingomyelinase
CDase	ceramidase
(F)FA	(free) fatty acid
ER	endoplasmic reticulum
TLC	thin layer chromatography
HPLC	liquid chromatography
HPTLC	high performance thin layer chromatography
LC-ESIMS/MS	liquid chromatography-electrospray ionization mass spectrometry
LC-NMR	liquid chromatography-nuclear magnetic resonance
CE-MS	capillary electrophoresis-mass spectrometry
IL-6	interleuchine-6
TNF-α	tumor necrosis factor-alpha
SPT	serine-palmitoyl transferase
TLR-4	toll-like receptor 4
LPS	lipopolysaccharides
NF-κB	nuclear factor kappa-light-chain-enhancer of activated B cells
JNK	c-Jun *N*-terminal kinases
SOCS	suppressor of Cytokine Signaling
SMPD	sphingomyelin phosphodiesterase
HFD	high fat diet
NAFLD	non-alcoholic fatty liver disease
T2DM	type 2 diabetes mellitus
HOMA-IR	homeostatic Model Assessment for Insulin Resistance
AST	aspartate Aminotransferase
ALT	alanine aminotransferase
LPC	lysophosphatidylcholine
SphK	sphingosine kinase
LacCer	lactosylceramide
dhSM	dihydrosphingomyelin
IR	ischemia-reperfusion
HIF1-α	hypoxia inducible factor 1-α
